# Abnormal Habits in Animals

**Published:** 1884-07

**Authors:** Felix L. Oswald


### ART. XV.—ABNORMAL HABITS IN ANIMALS.

BY FELIX L. OSWALD, M.D.

Abnormal habits in animals are nearly always a consequence of abnormal circumstances. In a controversy on original sin my opponent once tried to demonstrate the prevalence of natural depravity in the *primates* of the animal kingdom by mentioning the vitiated tastes of our quadrumanous relatives—their obscenity, their gluttony, and fondness for intoxicating drinks. But his argument only proved depravity in the keepers of the deluded four-handers. Monkeys with a penchant for alcoholic stimulants generally owe their foible to the machinations of some ancient mariner and the propagandism of vice which makes leisure on shipboard a moral curse. It is true that wild monkeys are sometimes caught by means of intoxicating baits; but, for such purposes, the taste of the tipple has to be disguised by a considerable percentage of saccharine admixtures. In captive animals the forcible administration of noxious stimulants now and then results in the development of a vitiated taste. Two Mexican miners, who used to torment a pet mountain sheep by catching it and stuffing its nose with snuff, suffered a just retribution when the resulting passion for nicotine made the bighorn an incorrigible tobacco-thief, who had no hesitation in forcing the door of an unguarded cabin, and upsetting tables and shelves in quest of the weed. In rare cases starvation may lead animals to the adoption of an unnatural diet. The sheep of Heligoland eke out an existence by munching fish-bones. The stall-fed dogs of China and Siam have learned to eat pumpkins, and there is no doubt that

*parrots* can become fond of dried beef. In Australia millions of parrakeets pick the offal of the beef-packeries, and even attack wounded or dying steers; and the same report comes from the Brazilian province of Entre Rios, where thousands of square leagues of the primeval forest have been turned into arid steppes that afford no sustenance to the frugivorous autochthones. Tree-fruits, and perhaps berries and nuts, I believe, made up the original *menu* of our Darwinian relatives; but in the sterile rock-hills of northern Africa many of them have become so used to an insect diet that they actually prefer it to other food. The Moor monkey (*Semnopithecus maurus*) robs orchards for the fun of the thing, and breaks off a dozen oranges for one he eats: but he never fails to swallow every butterfly, spider or caterpillar he can lay his hands on. According to Dr. Brehm the Abyssinian mantle-baboons eat even scorpions, which they catch and detail with two rapid grips. The East Indian macaques, too, are extremely fond of flies, and even their flea-hunts in the fur of an infested comrade seem to have an egotistical purpose, for every now and then the entomological investigator carries his fingers to his lips. American monkeys, on the other hand, care very little for such things. They are all forest-dwellers, and luxuriate in the original diet of their species. Only long captivity can change their predilections in that respect, though their fondness for nibbling sometimes tempts them to try their teeth on indigestible substances. F. J. Thompson, of the Cincinnati Zoo, once dissected a capuchin monkey who had died in convulsions that could be traced to no external cause, and found in his stomach a curiously twisted broom-corn ball. The little Brazilian had devoted his leisure to the collection and deglutition of every fragment of broom-furze which the keeper dropped in sweeping the cage.

There is a curious correlation between *truculence* and carnivorous habits. An exclusive bull-beef diet has imbued our North American redskins with all the instincts of beasts of prey; and, *vice versa*, it seems that the excitement (by cruel treatment, for instance) of vindictive passions can develop in all mammals an unnatural *blood-thirst*. The last Guicovar of Baroda kept, among other queer pets, a carnivorous horse, which once, in the presence of several British officers, knocked down a goat, and devoured her udder with all the ferocity of a famished wolf. A cross-examination of the hostler elicited the fact that the equine ogre had once been the property of a certain Rajah who had the reputation of being the most ill-tempered biped in northern Hindostan, and whose inhumanity had turned his nag into a werewolf. In cold winters instinct sometimes prompts certain four-handed animals, macaques especially, to indulge in such non-nitrogenous substances as fat bacon and tallow—in order to stifle tubercles, I conjecture, for no degree of hunger will tempt some of them to eat lean meat. Their *horror naturalis*, however, has nothing demonstrative about it; they will smell a piece of beef, cautiously lick it, perhaps, and then drop it, like so much indigestible stuff. Salt, on the contrary, they reject with every symptom of indignation, and I am sometimes tempted to suspect that the scientific clairvoyance of Dean Swift was not much at fault when he eulogized his children of nature for abstaining from saline condiments. A contemporary sage defends the practice as a physiological necessity, because, forsooth, deer and black cattle—*i. e.*, graminivorous brutes—are fond of visiting salt-licks; but our frugivorous relatives seem to attain eupepsia by other means, and our (probably abnormal) meat appetite would not alter the case, for the carnivora agree in taking their meat "straight." A Russian wolf, I believe, would starve for a considerable length of time rather than touch a piece of Russian salt beef. Yet the same monkeys that abhor salt, pepper and mustard are often ravenously fond of *onions*. It is either a consequence of necessitous circumstances, which, in southern Africa, for instance, compel millions of starving baboons to root for a living, or else Claude Bernard is right in classing onions with the comestibles rather than with the vegetable stimulants. He relates an anecdote of one Latour, a French 'longshoreman, who "earned from ten to twenty francs a day, gave alms and loaned money on interest, but slept at night in his basket, and subsisted on fourteen onions a day, which preserved him in excellent health and humor, but got him the nickname of *quatorze oignons*."

Dr. C. E. Page holds that the natural coat of a horse is a better protection against cold weather than the warmest horse-blanket; but it cannot be denied that in our hyperborean climate the natives of the lower latitudes soon learn to appreciate the value of artificial covers, though the attainment of that experience is not aided by any natural instinct. In houses where the temperature of cold winter nights is not counteracted by all-night fires, pet monkeys have to be tied up in their beds and swaddled like babies, or they will crawl out and freeze to death; but after one or two winter-campaigns they become, squirrel-like, fond of warm nests, and I once had a bonnet-macaque who supplemented his personal deficiency in animal warmth by catching a young poodle-dog and taking it to bed with him every night. Horses, too, though in their colthood they shudder at the mere touch of a woolen blanket, soon learn to value its calorific properties, and will stand stockstill all night for fear of losing the useful tegument.

And the study of the various species of four-handers in their native haunts would convince any unprejudiced observer that their alleged innate obscenity is an artificially acquired vice. I have studied their habits in Algiers and Central America, without ever seeing anything to justify the opinion that they are more salacious than their less hairy relatives, or that anything like an innate propensity prompts them to genesic aberrations. Dog-headed baboons, Moor monkeys, macaques, spider monkeys and capuchins can play in troops for hours together, without ever resorting to sexual pastimes. But it cannot be denied that captivity—especially in solitary confinement—makes some of them wonderfully and fearfully indecent. A Rhesus baboon, for instance, though his ancestry ranks high among the worthies of the Hindoo pantheon, is apt to contract habits that would scandalize even the tolerance of a Brooklyn congregation. A full-grown representative of that queer sect of saints, an almost hairless old male, named Bhunder-Beg, or Sahib Onki-Walla, was presented to Professor Gascar, of the "Zoological Park" of Marseilles; but the stockholders found themselves obliged, though with sincere regret, to send him back with the very next steamer. "I should like to keep him for the sake of your kind intention," Prof. G. wrote to the donor, "but it won't do; *with the sole exception of Petronius Arbiter*, your Bh., surnamed S. O. W., is the most obscene personage known to ancient or modern history."

